# Growth Temperature Influence on Lipids and Photosynthesis in *Lepidium sativum*

**DOI:** 10.3389/fpls.2020.00745

**Published:** 2020-06-04

**Authors:** Hamed Sattari Vayghan, Shahrzad Tavalaei, Armand Grillon, Léa Meyer, Gent Ballabani, Gaëtan Glauser, Paolo Longoni

**Affiliations:** ^1^Laboratory of Plant Physiology, Faculty of Sciences, Institute of Biology, University of Neuchâtel, Neuchâtel, Switzerland; ^2^Neuchâtel Platform of Analytical Chemistry, Faculty of Sciences, University of Neuchâtel, Neuchâtel, Switzerland

**Keywords:** photosynthesis, thylakoid membrane, membrane lipids, plastoquinone, temperature stress, electron transport

## Abstract

Temperature has a major impact on plant development and growth. In temperate climates, the seasonal temperature displays large variations that can affect the early stages of plant growth and development. Sessile organisms need to be capable of responding to these conditions, so that growth temperature induces morphological and physiological changes in the plant. Besides development, there are also important molecular and ultrastructural modifications allowing to cope with different temperatures. The chloroplast plays a crucial role in plant energetic metabolism and harbors the photosynthetic apparatus. The photosynthetic light reactions are at the interface between external physical conditions (light, temperature) and the cell biochemistry. Therefore, photosynthesis requires structural flexibility to be able to optimize its efficiency according to the changes of the external conditions. To investigate the effect of growth temperature on the photosynthetic apparatus, we followed the photosynthetic performances and analyzed the protein and lipid profiles of *Lepidium sativum* (cress) grown at three different temperatures. This revealed that plants developing at temperatures above the optimum have a lower photosynthetic efficiency. Moreover, plants grown under elevated and low temperatures showed a different galactolipid profile, especially the amount of saturated galactolipids decreased at low temperature and increased at high temperature. From the analysis of the chlorophyll *a* fluorescence induction, we assessed the impact of growth temperature on the re-oxidation of plastoquinone, which is the lipidic electron carrier of the photosynthetic electron transport chain. We show that, at low temperature, along with an increase of unsaturated structural lipids and plastochromanol, there is an increase of the plastoquinone oxidation rate in the dark. These results emphasize the importance of the thylakoid membrane composition in preserving the photosynthetic apparatus under non-optimal temperatures.

## Introduction

Plant metabolism must respond to daily and seasonal temperature variations. Photosynthesis, which is the main energetic process allowing plants to produce organic carbon, chemical energy, and reducing power, is no exception. Furthermore, the photosynthetic process is peculiar in the fact that uses light as the energy source of the primary photosynthetic reactions. As temperature differences affect the biochemical reactions but not the pigment excitation, the conversion of light energy into chemical energy needs to be finely tuned to be adapted to different temperatures (Yamori et al., 2014).

Light energy conversion requires a series of pigment–protein complexes, namely photosystem II (PSII), cytochrome b_6_f (Cyt*b_6_f*), and photosystem I (PSI). These complexes are functionally connected constituting an electron transport chain (Rochaix, 2011). The electrons obtained from the water-splitting reaction at PSII are transferred to plastoquinone (PQ), which is reduced to plastoquinol (reduced PQ). Reduced PQ diffuses into the membrane until Cyt*b_6_f*. This complex oxidizes the reduced PQ, splitting the two electrons obtained from the oxidation, one toward the plastocyanin and the other to a second PQ molecule. The electrons from plastocyanin are then transferred to PSI and from this complex to the final acceptor NADP via ferredoxin. The water-splitting and the cycle of the PQ during the electron transport increase the proton concentration in the thylakoid lumen generating a pH gradient across the membrane (ΔpH). ΔpH is used by the ATP synthase complex to produce ATP. The light absorption capacity of PSI and PSII reaction centers is extended by the light harvesting complex II (LHCII), a complex of trimeric and monomeric proteins binding chlorophylls and carotenoids that are capable to capture photons converting them into excitation energy. LHCII proteins can also act as dissipators of excess light energy by releasing part of the excitation energy as heat. Fine regulation of this energy dissipation has an important impact on plant growth and productivity (Kromdijk et al., 2016; Ware et al., 2016). The transmembrane ΔpH is the trigger of the thermal dissipation (Wraight and Crofts, 1970), the presence and abundance of the protein PsbS promote its induction (Li et al., 2000), while the xanthophylls, primarily lutein and zeaxanthin, are the main pigments acting as dissipators (Dall’Osto et al., 2010; Leuenberger et al., 2017). The processes allowing the dissipation of light excess as heat take collectively the name of non-photochemical quenching (NPQ).

The photosynthetic proteins involved in the electron transport chain are sensitive to changes in temperature. Prolonged low temperature results in the increase of the trimeric LHCII over monomeric LHCII in maize (Caffarri et al., 2005), and high temperature induces a change in the phosphorylation of the thylakoid proteins and by this also their localization in the thylakoid membrane subdomains (Rokka et al., 2000). Furthermore, the electron flux toward alternative pathways increases at suboptimal temperatures resulting in a lower efficiency of photosynthetic electron transport (Ivanov et al., 2012). These alternative electron routes, such as chlororespiration through the plastidic terminal oxidase (PTOX), water–water cycle, cyclic electron flow, and mitochondrial oxidative electron transport, may act collectively in reducing the excitation pressure on the photosystems and thus protecting the photosynthetic proteins from photodamage (Ivanov et al., 2012; Sunil et al., 2019).

Light reactions of the photosynthesis depend also on the lipids constituting the membrane in which the protein complexes are embedded. The membrane lipid composition influences PQ diffusion, and this may affect its functionality as electron transporter (Kirchhoff et al., 2002). The structural lipids of the thylakoid membrane are mostly galactolipids: Monogalactosyldiacylglycerols (MGDGs), representing around 50% of the total thylakoid lipids by weight, and digalactosyldiacylglycerols (DGDGs), constituting about 26%. Anionic lipids phosphatidylglycerol (PG) and sulfoquinovosyldiacylglycerol (SQDG) constitute most of the remaining lipid fraction of the thylakoid membrane (Dörmann, 2013). The temperature influences thylakoid membrane composition as well. At high temperature, the thylakoid membrane accumulates structural lipids with a higher degree of saturation (Falcone et al., 2004; Higashi et al., 2015). In addition to the structural lipids, the thylakoid membrane also contains antioxidant molecules such as tocopherols and plastochromanol (Mène-Saffrané et al., 2010; Spicher et al., 2016). These antioxidants have been shown to have an important role in the preservation of the photosynthetic apparatus during stress conditions such as high light (Rastogi et al., 2014), high temperature (Spicher et al., 2016), and their combination (Spicher et al., 2017). Furthermore, the chloroplast ultrastructure was also reported to change in response to temperature, for instance, following a temperature increase, the *Arabidopsis thaliana* chloroplast was reported to swell and the number and size of the internal lipid droplets, known as plastoglobules, was reported to increase (Zhang et al., 2010).

Here we investigate the impact of two growth temperatures, one above (30°C) and one below (10°C) the optimal growth temperature (22°C), on the photosynthetic apparatus of *Lepidium sativum* (cress). Cress is a fast-growing species belonging to the *Brassicaceae* family. We will focus on the differences in thylakoid membrane lipids and on the alternative pathways for the photosynthetic electrons as an adaptive strategy to reduce the excitation pressure and thus the damage of the photosynthetic apparatus.

## Materials and Methods

### Plant Growth and Stress Condition

Seeds of *L. sativum* were obtained from a local supplier. The seeds were put on soil and kept overnight at 6–8°C in the dark for stratification. The seeded pots were then transferred at 22°C under long day illumination (16 h L/8 h D, photosynthetic photon flux density in photoactive radiation PAR spectrum 86 μmol photons m^–2^ s^–1^) and allowed to germinate for 48 h. After germination, the plants were moved to 10°C or 30°C under the same light conditions, or maintained at 22°C, and grown for 5 additional days. Warm and cold conditions were produced within a FitoClima 600 (Aralab) climatic chamber. The length of the hypocotyl was measured manually for each plant. The leaf area per plant was calculated with ImageJ (NIH) measuring the green area of each plant from a top view picture using a scale for reference as previously described (Longoni et al., 2019). Five samples constituting the epigeal part of three plants were collected at the end of the growth to measure the average plant dry weight. For that, the samples were lyophilized for 120 h (FreeZone 2.5, Labconco, Kansas City, MO, United States) before weighing. To calculate the dry weight percentage over fresh weight, five samples per temperature, containing only the leaves collected from three plants, were weighted before and after 120 h of lyophilization.

### Photosynthetic Parameters

Each batch of plants was kept in the dark for at least 10 min before the measurements. Room temperature chlorophyll fluorescence was measured with a MF800 Fluorcam (Photon System Instrument, Czechia)^1^ employing a personalized light protocol (Pralon et al., 2020). The protocol is composed of blue light (470 nm) steps of 1 min with increasing intensity (35, 125, 315, 500, 690, and 875 μmol photons m^–2^ s^–1^ of PAR intensity). F_M_’ for each light intensity was measured with a saturating pulse at the end of the corresponding light step. After every light step, the actinic light was turned off for 10 s. During the first 2 s, far-red light was turned on to oxidize the photosynthetic electron transport chain. F_0_’ for each step was measured during the remaining part of the dark period. F_S_ is the steady-state fluorescence recorded at each light condition before the saturating light pulse. PSII quantum yield under light (ΦPSII) was calculated as ΦPSII = (F_M_’−F_S_)/F_M_’. The fraction of the open PSII centers (qL) was calculated with the following formula: qL = [(F_M_’−F_S_)/(F_M_’−F_0_’)]^∗^(F_0_’/F_S_) (Kramer et al., 2004). The non-photochemical energy dissipation was measured as NPQ = (F_M_–F_M_’)/F_M_’. The average fluorescence signal of each plant was used for the calculation of the photosynthetic parameters. Rapid chlorophyll fluorescence induction of PSII was measured on detached leaves with Plant Efficiency Analyser (M-PEA 2; Hansatech Ltd). The following protocol was used: after an initial saturating pulse (3000 μmol photons m^–2^ s^–1^, 700 ms, red light, dominant λ625 nm), the saturating light pulse was repeated after sequentially longer dark intervals (0.05, 4, 8, 12, 16, 20, and 24 s) for a total of eight pulses. After the eighth pulse, far-red light (20%) was turned on, and the leaf sample was submitted to a second series of saturating pulses separated by increasing time intervals (0.05, 0.1, 0.2, 0.4, 0.8, and 1.6 s) with continuous far-red light in between the pulses. For each saturating pulse, the parameters of the fast chlorophyll fluorescence curve were extrapolated by the M-PEA 2 software (Hansatech Ltd). The variable fluorescence at 3 ms (*V*_J_) and the Fo normalized over the maximal fluorescence (F_O_/F_M_) were analyzed by plotting them over the time interval between the pulses.

### Protein Analysis

Plant samples were ground to a fine powder in a 1.5 ml plastic tube with glass beads in an Ivoclar Vivadent shaker (Silamat). The proteins were extracted adding 10 μl per mg FW of lysis buffer [100 mM Tris–HCl, pH 8.5, 2% SDS, 10 mM NaF, 0.05% of protease inhibitor cocktail for plants (Sigma)], vortexing and incubating the sample at 37°C for 30 min. Plant debris was removed by centrifugation (5 min at 16,000 × *g*) at room temperature. The samples were diluted four times in water and supplemented with gel loading buffer (50 mM Tris–HCl, pH 6.8, 100 mM dithiothreitol, 2% SDS, 0.1% bromophenol blue, 10% glycerol). After denaturation at 75°C for 10 min, an aliquot of 10 μl of the diluted sample was loaded in the gel. The protein separation was performed following the standard SDS-PAGE protocol in a 12% acrylamide bis acrylamide (37.5:1) gel (Laemmli, 1970). The proteins were transferred on a nitrocellulose membrane, stained for 30 s with amido black solution (15% isopropanol, 10% acetic acid, 0.1% w/v amido black), and destained with a destaining solution (40% ethanol, 10% acetic acid). An image of the stained membrane was taken for the quantification of the total proteins in each lane. The membrane was blocked with 5% milk in TBS-0.05% Tween and then decorated with the following antibodies: anti-Lhcb2 (Agrisera, AS01 003), anti-Lhcb1 (Agrisera, AS01 004), anti-D1 (PsbA) (Agrisera, AS05 084), anti-PetC (Agrisera, AS08 330); anti-PsaD (Agrisera, AS09 461), anti-RbcL (Agrisera, AS03 037A); a secondary antibody anti-rabbit conjugated with HRP (Merk) was used to allow the detection of the protein by chemioluminescence (ECL). The ECL signal was recorded with a CCD camera (Amersham Imager 600 Amersham Biosciences, Inc.), set on automatic detection time. Band intensity was measured with ImageQuant TL 8.1 Software (GE Healthcare Life Sciences). The obtained values were normalized over the sum of the amido black band intensities measured with the same software using a minimum profile subtraction method. The protein extraction was performed on leaf samples harvested during at least two independent biological replicates (9 total samples for PsbA and PsaD, 6 samples for RbcL and PetC, 5 for Lhcb2 and 4 for Lhcb1). For PTOX detection (Agrisera, AS16 3692), the membrane was blocked in 6% BSA in TBS-0.05% Tween; the same solution was used for the preparation of the primary and secondary antibody dilutions.

### Pigments and Lipid Profiling

The sample used for chlorophyll quantification and lipidomic profiling is constituted of at least two leaves collected from two different plants grown at the same temperature. Chlorophyll extraction was performed in 80% acetone by adding 10 μl of solvent per mg of FW of leaf sample. The sample was further diluted twofold in 80% acetone before measuring the absorbance at multiple wavelengths (470, 646, 663 nm). The quantification of chlorophyll *a* and *b* was performed according to Lichtenthaler and Wellburn (1983). The measured chlorophyll concentration was adjusted to the dry weight based on the average leaf water content measured for each growth temperature. The measure was repeated on 15 samples from different plants obtained from five independent experiments. Extraction of the lipid fraction was performed on 12 samples from different plants obtained from three independent experiments. In brief, after grinding, the lipids were extracted in 10 μl of tetrahydrofuran:methanol 50:50 (v/v) per mg of FW, plant debris was separated by centrifugation (3 min, 14,000 × *g*), and the supernatant was carefully transferred to an HPLC vial. The lipids were separated by ultra-high pressure liquid chromatography and identified by a coupled atmospheric pressure chemical ionization-quadrupole time-of-flight mass spectrometry (UHPLC-APCI-QTOF-MS) as previously described (Spicher et al., 2016). Lipids were separated on a reverse-phase Acquity BEH C18 column (50 × 2.1 mm, 1.7 μm) maintained at 60°C. The elution was as follows: solvent A = water; solvent B = methanol; 80–100% B in 3 min, 100% B for 2 min, re-equilibration at 80% B for 2.0 min. The flow rate was 0.8 ml min^–1^ and the injection volume was 2.5 μl. Data were acquired using MassLynx version 4.1 (Waters) and further processed with QuanLynx (Waters). Compound identity was determined based on reference standards that were also used for the quantification curve (Spicher et al., 2016). Violaxanthin and neoxanthin could be resolved neither in the chromatographic nor in the mass dimensions under the conditions employed; therefore, the measured peak corresponds to the sum of these compounds. The same applies for lutein and zeaxanthin. MGDG 36:6, 36:5, 36:4, 34:6, 34:5, 34:4 and DGDG 36:6, 36:5, 36:4, 36:3, 34:6, 34:3, 34:2, 34:1 identities were confirmed comparing the peak of the extract with a standard plant MGDG mix and DGDG mix (Avanti Polar Lipids). The calibration curves of MGDG 36:6, MGDG 34:6, DGDG 36:6, and DGDG 34:6 were used for the quantification of all MGDG 36:x, MGDG 34:x, DGDG 36:x and DGDG 34:x, respectively (Supplementary Figure S1). To avoid any bias due to signal saturation in the quantification of the most abundant DGDGs, the samples were measured a second time following a tenfold dilution in tetrahydrofuran:methanol 50:50 (v/v). The values obtained by the calibration curves were corrected by the percentage of each molecule composing the standard mix, as reported by the manufacturer (Avanti Polar Lipids). The molecules not detected in the standard mix were tentatively characterized based on m/z and retention time characteristics, which vary proportionally to the degree of acyl chain length and saturation (Li et al., 2008).

### Statistical Analysis

The normal distribution of the residuals of each data set was tested before any other statistical analysis. If this assumption was met, an ANOVA model was utilized; otherwise, a Kruskal–Wallis rank sum test was performed. If the results were significant, we used *post hoc* Tukey’s HSD test for multiple comparisons. The reported *p*-values were obtained with the latter. The calculations were performed with RStudio (Version 1.2.5019 RStudio Inc).

## Results

### Growth Temperature Affects Photosynthetic Efficiency

**FIGURE 1:**
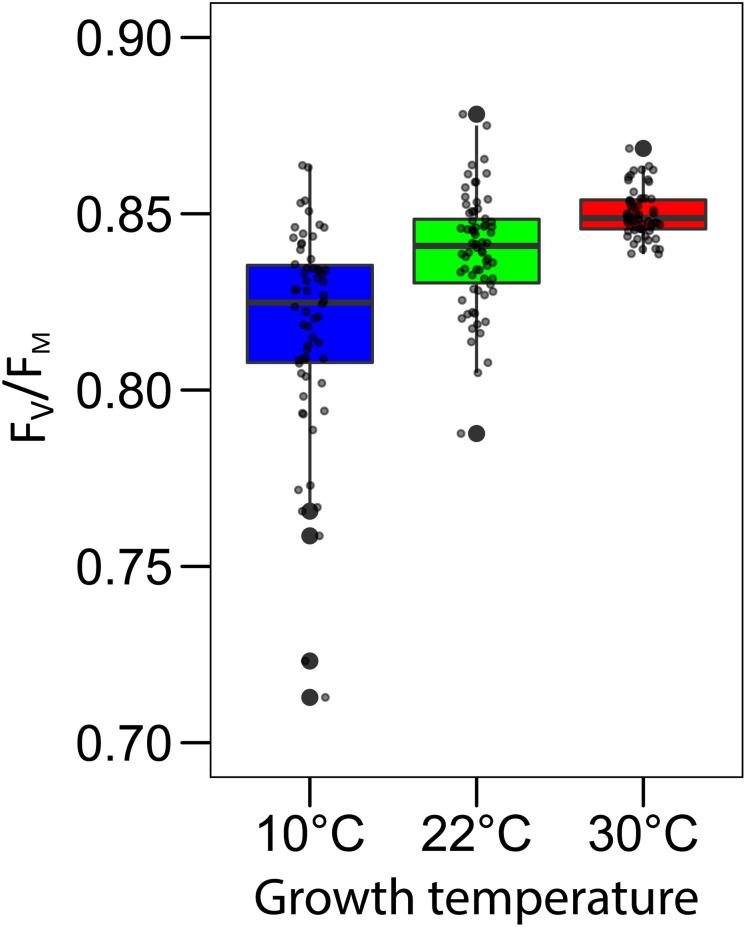
Growth temperature has a minimal impact on the Photosystem II maximum efficiency. The maximum PSII quantum yield was measured in trays containing 20–24 plants, grown at three different temperatures (10, 22, and 30°C) for 5 days. Before measuring, the plants were incubated 10 min in the dark at room temperature. Gray points represent the average value for each single plant. In the box plots, the lower and upper hinges correspond to the first and third quartiles (the 25th and 75th percentiles). The whiskers extend from the hinge to the farthest value no further than 1.5 times the distance between the first and third quartiles from the hinge. Farther points are considered as “outliers” and plotted individually as solid black points.

**FIGURE 2:**
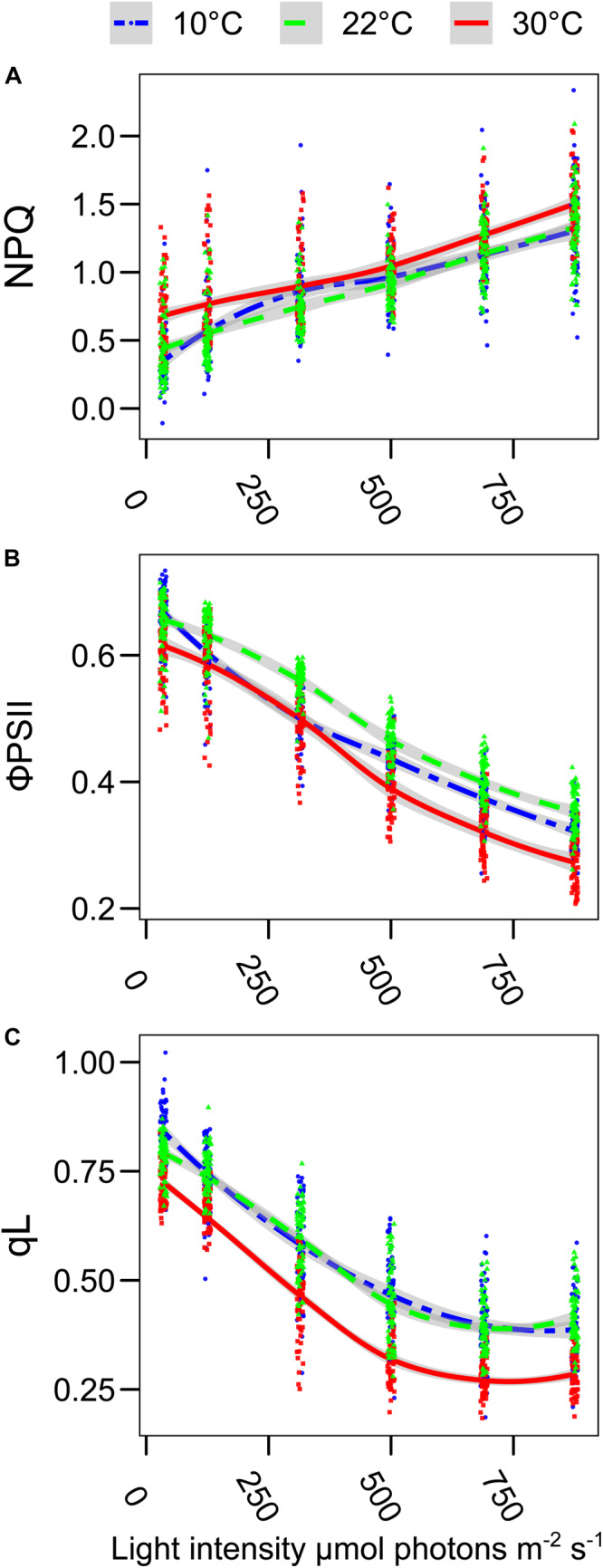
Growth temperature affects the photosynthetic electron transport chain in *Lepidium sativum*. The room temperature fluorescence was measured on trays with 20–24 plants, grown at the three different temperatures (10°C blue, 22°C green, and 30°C red). Prior to the measurement, the plants were incubated 10 min in the dark at room temperature and directly used for the measure of the chlorophyll *a* fluorescence kinetic. **(A)** Level of thermal dissipation measured as NPQ for the three growth conditions after 1 min of exposure to increasing light intensity. **(B)** Photosystem II quantum yield (ΦPSII) measured at the end of each 1-min light intensity step. **(C)** Fraction of the open PSII reaction centers (qL) after 1 min of exposure to increasing light intensities. The light intensities are expressed as μmol photons m^–2^ s^–1^ of blue light (470 nm), and the same scale is used for the three graphs. The points were fitted with a local regression algorithm based on a polynomial quadratic function to visually represent the data distribution in function of the light intensities with a continuous error estimate. The fit is shown for the three growth conditions (10°C, blue double dashed line, 22°C green dashed line, and 30°C red continuous line). The standard error of the interpolation is shown as a gray area around the curve. All the data points from the 30°C growth are statistically different from the 22°C control conditions (*Post Hoc* analysis, *p* < 0.0001). The three parameters were measured simultaneously, and they originated from two independent experimental replicates (30°C, *n* = 60; 22°C, *n* = 70; 10°C, *n* = 62).

The plants of *L. sativum* grown at different temperatures had a slightly different morphology. The leaf area was smaller in the plants grown at 10°C and 30°C compared with the control condition. The hypocotyl was longer in the plants grown at 30°C, while there was no significant difference in the other two conditions. However, both the plants grown at 10°C and 30°C produced less dry biomass per plant (Supplementary Figure S2). The entire pot, containing 24 plants, was analyzed by chlorophyll fluorescence to determine the impact of the different growth temperatures on the overall functionality of the photosynthetic electron transport chain. The first parameter observed was the maximal quantum yield of the photosystem II (F_V_/F_M_), a decrease in this value would suggest the presence of persistent damage on PSII. The maximum quantum yield was slightly lower in the plants grown at 10°C (*p* < 0.0001) compared to the other two growth temperatures (Figure 1). However, the average value of 0.83 measured at 10°C, which is PSII efficiency reported for healthy plants, suggests that there was no major damage during the growth at this suboptimal temperature (Maxwell and Johnson, 2000). The negligible effect on the maximum yield of PSII allowed investigating further the photosynthetic activity and adaptation under increasing actinic light intensity. Chlorophyll *a* fluorescence was measured at 22°C by red flashes while stepwise increasing the actinic light intensity. The use of a saturating pulse at the end of each light step allowed the calculation of the fraction of the energy dissipated as heat (NPQ) at each light intensity (Caffarri et al., 2005). Plants grown at 30°C had a higher NPQ value compared to those grown under control temperature (Figure 2A). This effect was evident already at the lower light intensity tested (35 μmol photons m^–2^ s^–1^) (*p* < 0.0001 compared to both 10°C and 22°C), a condition in which the NPQ of the plants grown at 10°C was statistically lower than the control (*p* = 0.0005). Only at 315 μmol photons m^–2^ s^–1^, the plants grown at 10°C appear to have a slightly higher NPQ compared with the control condition (*p* = 0.11); otherwise, there was no statistically significant difference between these two conditions. The changes in the NPQ are symmetrically reflected in the quantum yield of photosystem II photochemistry (ΦPSII, Figure 2B). The plants grown at 30°C had constantly a lower yield of PSII compared to the control growth temperature. ΦPSII of those grown at 10°C was also lower, especially at 125 and 315 μmol photons m^–2^ s^–1^ (*p* < 0.0001). While at higher light intensities, ΦPSII difference between the plants grown at 10°C and those at 22°C became smaller. Therefore, at the higher light intensities tested, the plants grown at 10°C had a higher ΦPSII compared to the plants grown at 30°C. Analysis of the fraction of open PSII centers (qL) revealed that the plants grown at 30°C had a steady increase in the fraction of closed PSII as measured by the qL parameter (Baker, 2008). Surprisingly, this is not the case for the plants grown at 10°C, which have a similar qL level compared to the control temperature (22°C) (Figure 2C). Since these differences may underlay a change in the organization of the electron transport chain, we further analyzed the protein and the lipid composition of the plants grown at the different temperatures.

### Effect of Growth Temperature on Major Thylakoid Pigments and Proteins

**FIGURE 3:**
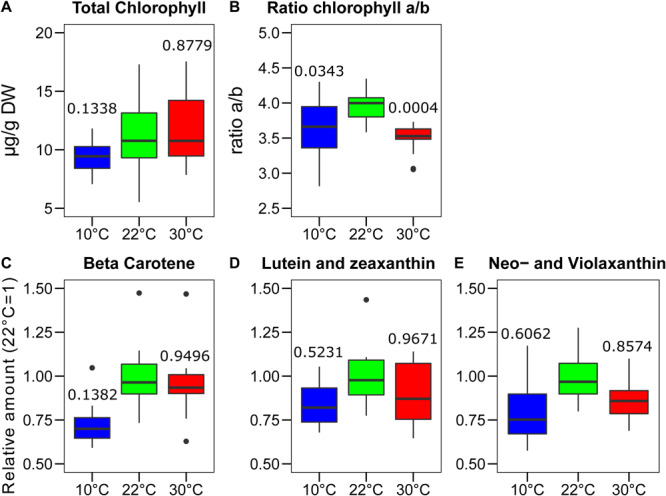
Changes in pigments content after the growth of *Lepidium sativum* at different temperatures. The chlorophyll content was measured in a sample constituted of two leaves collected from different plants grown in the same condition. **(A)** Content in total chlorophyll measured after the growth at three different temperatures normalized on the leaf dry weight. **(B)** Chlorophyll *a/b* ratio measured in the same samples shown in **(A)** (*n* = 15). Carotenoids analysis was performed by chromatography coupled with mass spectrometry (*n* = 11). The values are reported in terms of differences from the average value at 22°C for **(C)** Beta-carotene, **(D)** Lutein and zeaxanthin, and **(E)** neoxanthin and violaxanthin. The carotenoids in the blots **(D)** and **(E)** were indistinguishable in time or *m*/*z* and therefore are plotted as the sum of the two. In the box plots, the lower and upper hinges correspond to the first and third quartiles (the 25th and 75th percentiles). The whiskers extend from the hinge to the farthest value no further than 1.5 times the distance between the first and third quartiles from the hinge. Farther points are considered as “outliers” and plotted individually. The *p*-value derived from a *post hoc* test comparing the data with the value at 22°C is shown above each box.

The stoichiometry of the different photosynthetic complexes of the thylakoid membrane, namely the two photosystems (PSI and PSII), the cytochrome *b_6_f* (Cyt*b_6_f*), and the light harvesting complex II (LHCII), may vary upon acclimation of the photosynthetic machinery to the environment. To test the impact of the growth temperature on the organization of the major thylakoid proteins, the total chlorophyll content was measured along with the chlorophyll a to *b* ratio. This gives an indication of the ratio between the LHCII antenna complex, enriched in chlorophyll *b*, and the two photosystems, which have a higher chlorophyll *a/b* ratio. The chlorophyll concentration was adjusted to the average calculated dry mass of the sample, which corresponds to 7% at 22°C and 8% both at 30 and 10°C of the respective fresh weight (*n* = 5). The chlorophyll concentrations of the plants grown at the three temperatures differed a little. Plants grown at 10°C had a slightly lower chlorophyll concentration compared to those grown at 30°C (*p* = 0.0626, *n* = 15) and at 22°C (*p* = 0.1338, *n* = 15) (Figure 3A). A clear difference was instead observed by comparing the chlorophyll *a/b* ratio. In both the plants grown at 30°C and at 10°C, the a/b ratio was significantly lower than the one observed at control temperature (22°C–30°C, *p* = 0.0004, *n* = 15; 10°C–22°C, *p* = 0.0343, *n* = 15) (Figure 3B). This suggests that the relative amount of chlorophyll *b* rich proteins, such as LHCII, increased in the plants grown in both non-optimal temperatures. The carotenoids were quantified by mass spectrometry (UHPLC-APCI-QTOF-MS). For the carotenoids analyzed, we observed a general trend toward lower accumulation at 10°C compared to the other two growth temperatures. However, these differences appeared not to be statistically significant. β-carotene was the compound showing the most consistent difference among biological replicates when comparing plants grown at 10°C with those grown at the control temperature of 22°C (*p* = 0.1382, *n* = 11) (Figure 3C). Other carotenoids associated with the photosynthetic proteins, instead, do not show a differential level of accumulation at the different temperatures; this is the case for lutein and zeaxanthin and for the combination of neoxanthin and violaxanthin (Figures 3D,E).

**FIGURE 4:**
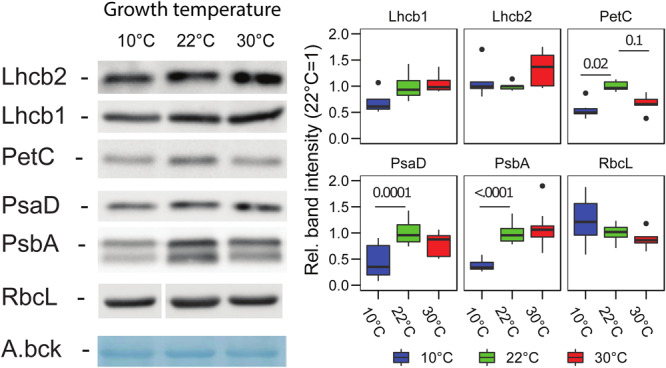
Photosynthetic protein accumulation in *Lepidium sativum* at different growth temperatures. Representative proteins for the complexes of the electron transport chain, PSII (PsbA), PSI (PsaD), Cytb_6_f (PetC), and LHCII (Lhcb1, Lhcb2) were analyzed by immunodetection in samples normalized on fresh weight. A representative blot is shown for each protein along with a representative band of the total transferred proteins stained with amido black (A.bck) as a protein loading control. The box plots show the band intensity, normalized over the total amido black lane staining, compared to the control condition (22°C) (average intensity 22°C = 1). PSII (PsbA/D1) (*n* = 9), PSI (PsaD) (*n* = 9), Cytb_6_f (PetC) (*n* = 6), LHCII (Lhcb2) (*n* = 5), Lhcb1 (*n* = 4), and RbcL (*n* = 6). In the box plots, the lower and upper hinges correspond to the first and third quartiles (the 25th and 75th percentiles). The whiskers extend from the hinge to the farthest value no further than 1.5 times the distance between the first and third quartiles from the hinge. Farther points are considered as “outliers” and plotted individually.

The changes in the accumulation of the major photosynthetic complex were estimated by immunodetection of the subunits of each complex in total protein extracts. Interestingly, it appeared that growth at 10°C, compared to the control at 22°C, results in a lower accumulation, on a total protein basis, of PSII (PsbA/D1) (*p* < 0.0001, *n* = 9), PSI (PsaD) (*p* = 0.0001, *n* = 9), Cyt*b_6_f* (PetC) (*p* = 0.02, *n* = 6). For the trimeric LHCII (Lhcb2 and Lhcb1), the reduction was milder so that we did not observe a significant difference in the detected protein compared to the 22°C condition (Lhcb1 *p* = 0.29, *n* = 4; Lhcb2 *p* = 0.82, *n* = 5) (Figure 4). At 30°C, the only significant difference observed by immunodetection was for PetC of the Cyt*b_6_f*, which was reduced similarly to the 10°C condition (10°C–30°C, *p* = 0.76; 22°C–30°C, *p* = 0.1) (Figure 4).

### Temperature-Dependent Changes in the Lipids of the Thylakoid Membrane

**FIGURE 5:**
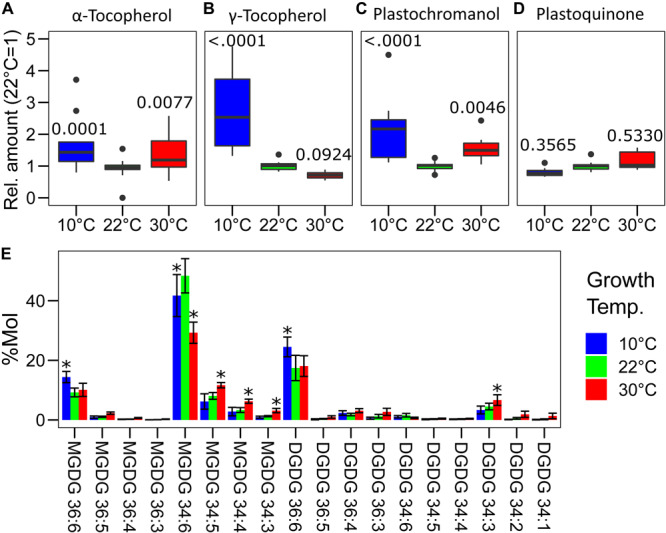
Profile of the galacto- and prenyl-lipids in *Lepidium sativum* grown at different temperatures. Total lipids were extracted from pooled leaf samples, separated by liquid chromatography and identified by mass spectrometry. Eleven leaf samples from three independent growth replicates were used. Relative concentration of the principal antioxidants of the thylakoid membrane **(A)** α-tocopherol, **(B)** γ-tocopherol, **(C)** plastochromanol, and its precursors **(D)** plastoquinone, the latter also serves as an electron carrier in the electron transport chain. In the box plots, the lower and upper hinges correspond to the first and third quartiles (the 25th and 75th percentiles). The whiskers extend from the hinge to the farthest value no further than 1.5 times the distance between the first and third quartiles from the hinge. Farther points are considered as “outliers” and plotted individually. The *p*-value derived from a *post hoc* test comparing the data with the value at 22°C is shown above each box. **(E)** Profile of the monogalactosyldiacylglycerols (MGDG) and digalactosyldiacylglycerols (DGDG) expressed as % molecules over the total of the measured galactolipids. The compounds are listed by the length and degree of saturation of the acyl chains, and significant differences from the control temperature 22°C (*p* < 0.05) are highlighted with an asterisk (*n* = 11).

Alpha-tocopherol and plastochromanol, major antioxidants present in the thylakoid membrane, were measured by mass spectrometry following chromatographic separation (Mène-Saffrané et al., 2010; Spicher et al., 2017; Figure 5). The amount of α-tocopherol, on a dry weight basis, was higher in both non-optimal temperatures compared to the plants grown at 22°C (10°C–22°C, *p* = 0.0001; 30°C–22°C, *p* = 0.0077; *n* = 11). The difference between 10 and 30°C was not statistically significant for this compound (*p* = 0.4834) (Figure 5A). Similarly, plastochromanol concentration was higher, 1.5-fold to twofold, in both temperature conditions (10°C–22°C, *p* < 0.0001; 30°C–22°C, *p* = 0.0046), with a tendency toward a greater amount at 10°C compared to 30°C (*p* = 0.1061) (Figure 5C). The accumulation of the two precursors of these molecules, γ-tocopherol and PQ, was measured as well. Despite a large difference between replicates, the concentration of γ-tocopherol was found to be higher at 10°C and slightly lower at 30°C compared to the control temperature (10°C, *p* < 0.0001; 30°C, *p* = 0.0924) (Figure 5B). This may suggest that at 10°C there could be an incomplete conversion of γ-tocopherol into α-tocopherol as vegetative tissues do not normally accumulate γ-tocopherol (Abbasi et al., 2007). This conversion may be possibly faster at 30°C, and this leading to the lower accumulation was observed. On the contrary, the amount of PQ was not significantly different in the three temperatures tested, even if a tendency toward higher PQ concentrations was observed in the plants grown at 30°C and toward lower concentrations in those grown at 10°C creating a significant difference between the two conditions (10°C–30°C, *p* = 0.0564) (Figure 5D).

The saturation degree of the lipids composing the biological membrane is known to change depending on temperature (Falcone et al., 2004). This is also the case for the major structural lipids of the thylakoid membrane that are monogalactosyldiacylglycerols (MGDG) and digalactosyldiacylglycerols (DGDG) (Block et al., 1983). The growth temperature had a wide impact on the profile of the galactolipids (Figure 5E). At 10°C, long-chained unsaturated mono- and digalactolipids (36:6) were relatively more abundant compared to the control temperature. The effect was opposite for the plants grown at 30°C in which there was a higher proportion of galactolipids with 34 carbons and particularly of those which are more saturated (34:3, 34:2), compared to the control condition at 22°C (Figure 5E). A specific difference was observed for MGDG 34:6, which is the most abundant galactolipid; its level was maximal in the plants grown at 22°C but lower at both 10 and 30°C (Figure 5E). Also, the MGDG/DGDG ratio was different in the plants grown at non-optimal temperatures. In fact, over the total of the measured galactolipids, the MGDG fraction was 72 ± 4% in the control condition (22°C) but it decreased to 67 ± 4% and 63 ± 3% at 10 and 30°C, respectively (10°C–22°C, *p* = 0.0184; 30°C–22°C, *p* < 0.0001; *n* = 11). Furthermore, there was also a different accumulation of the galactolipids originating from the endoplasmic reticulum (ER), containing two 18 carbon acyl chains (C18/C18), and those of plastid origin containing one 18 carbon chain and the second with 16 carbons (C18/C16) (Boudière et al., 2014). Considering the molar fraction of the measured galactolipids, in control condition (22°C), 32 ± 5% had two acyl chains of 18 carbons. In the cress plants grown at 10°C, the fraction of the C18/C18 galactolipids increased to 43 ± 5%, significantly higher than in the control condition (*p* < 0.0001, *n* = 11). An intermediate state was observed at 30°C with C18/C18 galactolipids representing 38 ± 3% of the total, differing from the cress grown at 22°C (*p* = 0.0092, *n* = 11) and at 10°C (*p* = 0.0214, *n* = 11). The average number of desaturations per molecule of the measured galactolipids was significantly lower (*p* < 0.0001, *n* = 11) in the plants grown at 30°C (5.1 ± 0.2) compared to the control condition (5.5 ± 0.1) and to the 10°C temperature (5.7 ± 0.1). The difference in the average number of desaturations per molecule was bigger considering only DGDGs, and the average number of desaturations per molecule at 22°C was 5.09 ± 0.2, significantly higher at 10°C (5.44 ± 0.2; 10°C–22°C, *p* = 0.0123) and lower at 30°C (4.62 ± 0.4; 30°C–22°C, *p* = 0.0009).

### Plastoquinone Oxidation Is Altered at Low Temperature

**FIGURE 6:**
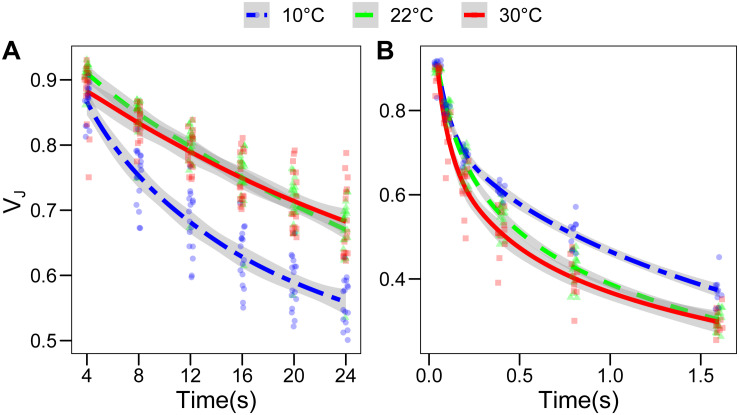
Fluorescence relaxation in the dark is faster in *Lepidium sativum* grown at low temperature. The fluorescence value measured at 3 ms during a 700 ms saturating pulse was normalized over the maximal fluorescence to obtain the *V*_J_ parameter. This parameter was used as a proxy of the redox state of the photoactive plastoquinone pool. **(A)** Evolution of the *V*_J_ parameter upon sequential incubations of the sample in the dark, for the three growth conditions. The points were fitted with a local regression algorithm based on a logarithmic function to visually represent the data distribution in function of the time with a continuous error estimate (10°C, blue double dashed line, 22°C green dashed line, 30°C red continuous line). The standard error of the interpolation is shown as a gray area around the curve. **(B)** Evolution of the *V*_J_ parameter upon sequential incubation with far-red light exciting preferentially the photosystem I. The statistical significance of the difference between non-overlapping error areas of the interpolation was confirmed by a *post hoc* test (*p* < 0.001) (10°C, *n* = 10; 30°C, *n* = 11; 22°C, *n* = 16). Different *X* and *Y* axes are used in **(A)** and **(B)** to highlight the differences between the growth temperatures.

The alteration of the lipid composition may have a functional impact on electron transport and on the PQ dynamics. We therefore assessed the re-oxidation kinetics of the photoactive PQ pool using an indirect method based on PSII fluorescence induction curve. It has been shown that the fluorescence level at 3 ms (Fj) correlates with the redox state of the Q_B_ site of photosystem II, and the latter is in equilibrium with the photoactive PQ pool (Tóth et al., 2007). Therefore, the relative fluorescence at 3 ms (*V*_J_) and its kinetics after a dark or far red-light period of increasing length were taken as a proxy of the PQ oxidation over time. In the dark, the PQ oxidation is performed by electron pathways alternative to the photosynthetic electron transport chain, mostly via the plastid terminal oxidase (PTOX) (Pralon et al., 2020). Conversely, far-red will activate PSI allowing a photochemical oxidation of PQ (Rochaix, 2011). As expected, PQ oxidation under far-red light was faster than the oxidation observed in the dark, PTOX being a much slower oxidizer than PSI (Tóth et al., 2007). The PQ oxidation in the dark, as inferred by the *V*_J_ parameter, was faster in the plants grown at 10°C compared to both other conditions (*p* < 0.001), which, on the contrary, did not differ significantly (Figure 6A). When the oxidation of the photoactive PQ pool was performed by the electron transport chain, exciting PSI with far-red light, the plants grown at 30°C showed a faster initial re-oxidation, up to 0.4 s (*p* = 0.0513), while further followed the same kinetics as the plants grown at 22°C. For the plants grown at 10°C, the effect was reversed; in fact, the kinetics up to 0.2 s were identical to the plants grown at 22°C, while the following re-oxidation kinetics appeared to be slower, so that the *V*_J_ was significantly higher at the 0.4 and 0.8 s time points (*p* = 0.0005 and *p* = 0.0015, respectively; Figure 6B). The fluorescence in the dark, before the saturating pulse (F_O_) calculated by the M-PEA instrument, does also decline with increasing time intervals between the pulses. However, the F_O_ value normalized over the F_M_ does not show the same difference between the samples as observed for *V*_J_ (Supplementary Figure S3), suggesting that the two parameters are partially independent.

## Discussion

Temperature is a challenge for the photosynthetic apparatus, affecting enzymatic kinetics, as well as membrane dynamics and organization (Yamori et al., 2014). It is therefore essential for the plant to adopt strategies to protect its photosynthetic apparatus while maximizing light utilization at the non-optimal temperature condition. Here we explored the response on the early growth of a fast-developing species: *L. sativum*, which belongs to the same family, *Brassicaceae*, as model organism *A. thaliana* and important crop species.

The total chlorophyll concentration displayed minor differences compared with those previously reported using other plant species that were exposed to high and low temperature (Grimaud et al., 2013; Spicher et al., 2016). This is possibly because the temperatures employed in this experiment were milder, and therefore, a strong detrimental stress to the photosynthetic apparatus was avoided. This is in line with the limited effect on the maximum quantum yield of PSII observed in the plants grown at 10°C (Figure 1). However, both the plants grown at 10 and 30°C showed an alteration in the measured photosynthetic parameters compared to the control temperature (Figure 2). It has to be underlined that all the photosynthetic measurements were performed at 22°C, i.e., the temperature at which the control plants were grown, and it is therefore not surprising that plants grown at the same temperature are the best performers at this temperature. The average PSII redox state under light was inferred from the qL parameter, representing the probability of a photon to be transferred to an open PSII reaction center in an interconnected lake model (Kramer et al., 2004). Surprisingly, for the plants grown at 30°C, the qL was smaller compared to the plant grown at the two other temperatures (Figure 2). This result is consistent with the protein amount, as the plants grown at 30°C appear to have a lower level of the Cyt*b_6_f* complex (PetC), which is the rate limiting step of the electron transport chain under moderate light condition and in the presence of CO_2_ (Rochaix, 2011). This downregulation has been hypothesized to serve as a protective mechanism for the photosystems (Krieger-Liszkay et al., 2000). In this regard, the specific relative loss of Cyt*b_6_f* at 30°C may be part of the regulation mechanism to protect PSI challenged by the high temperature, by slowing down the electron flow toward PSI, while PSII repair cycle and the higher constitutive NPQ maintain PSII efficiency (Yamane et al., 1997). At 10°C, all the three complexes involved in the electron transport appear to be diminished to a similar extent compared to the control temperature. Therefore, there should be no effect on the relative electron transport capacity per PSII. Consistently, the fraction of open PSII reaction centers (qL) in 35 to 875 μmol photons m^–2^ s^–1^ of PAR intensity range is comparable to the control (Figure 2C). Another protective mechanism of PSII is the thermal dissipation of the light excess (NPQ), which also appears to be induced in the plants grown at 30°C that display a stronger thermal dissipation component already at the lowest light intensity used in the fluorescence measurement (Figure 2A). The growth at lower temperature results in a minor change of the thermal dissipation at moderately high intensity; however, the NPQ at higher light intensities (above 500 μmol photons m^–2^ s^–1^) was similar to the NPQ of the plants grown at control temperature (22°C). As NPQ induction is dependent on the transmembrane ΔpH (Wraight and Crofts, 1970), this observation suggests that plants grown at higher temperature could be less efficient in the dissipation of the ΔpH, or alternatively that the components of the NPQ machinery are over-accumulated in these plants. However, PsbS, which is the protein responsible for the fast NPQ component (Li et al., 2000), was reported not to respond to temperature as it is not induced in low temperature (Rorat et al., 2001; Norén et al., 2003). Furthermore, the *PSBS* gene expression was not altered upon changes in the growth temperature, neither by increasing nor by lowering it, in *A. thaliana* (Kilian et al., 2007). In the plants grown at the higher temperature, there was no increase in the concentration of the two xanthophylls, lutein and zeaxanthin, which are directly involved in the dissipation of the chlorophyll excitation energy, compared with the control (Figure 3; Leuenberger et al., 2017). Therefore, a tentative explanation for the higher NPQ observed in the plants grown at 30°C is the aggregation of LHCII (Tang et al., 2007); this effect should produce a constitutive energy dissipation that would explain the quenching detected already at low light intensity. A relative increase of LHCII over the photosystems would also explain the lower chlorophyll *a/b* ratio (Figure 3B). A tendency over a higher Lhcb2 accumulation was observed at 30°C (*p* = 0.14, Figure 4). The lack of a quantitative confirmation of this increased accumulation by immunodetection may be explained by an interference of the N-terminal phosphorylation of the antenna on the protein recognition by the antibodies (Longoni et al., 2015). Indeed, the Lhcb2 protein tends to be more phosphorylated when plants were grown at 30°C (Supplementary Figure S4). The level of NPQ affects also directly the quantum yield of PSII (ΦPSII). The plants grown at 30°C induced the most NPQ and had a lower ΦPSII in all the light conditions tested (Figure 2B). The factor limiting ΦPSII in cress grown at low temperature, on the contrary, does not appear to be the NPQ. Most likely in this case unrepaired damage on PSII is responsible for the lower efficiency (Grimaud et al., 2013). This hypothesis would be consistent with the slightly lower F_V_/F_M_ value measured in the plants grown at lower temperature (Figure 1). This shows that, despite both un-optimal temperatures affecting photosynthesis, the response to the temperature change is different, and not necessarily symmetrical, between lower and higher temperatures.

Membrane lipid composition, including galactolipids of the thylakoid membrane, varies in response to temperature (Falcone et al., 2004; Spicher et al., 2016). As expected, we observed a tendency toward the accumulation of long-chained unsaturated galactolipids (36:6) at 10°C, while the opposite (i.e., accumulation of 34 acyl-carbons and more saturated lipids) was true at higher temperature. This change is most likely necessary to have a similar level of membrane fluidity at different temperatures (Barber et al., 1984). The average number of desaturations per acyl chain in the measured galactolipids was significantly higher at 30°C and lower at 10°C compared to 22°C. The mode by which galactolipid desaturation influences the thylakoid membrane fluidity is not yet defined unambiguously. However, the level of galactolipid saturation does change in different plant species in response to a temperature shift (Falcone et al., 2004). Consistently, modification of the level of lipid saturation by knocking down or overexpressing individual fatty acid desaturases results in an altered resistance of the plants to extreme temperatures (Penfield, 2008). This suggests that the differences observed in the acyl chain saturation are part of cress acclimation to growth temperature. At both non-optimal temperatures, the cress accumulated a larger proportion of MGDG and DGDG originating from the ER (i.e., containing two acyl chains at 18 carbons (C18/C18)] over the galactolipids of plastid origin (i.e., containing one acyl chain of 18 carbons and one of 16 carbons (C18/C16)] (Figure 5; Boudière et al., 2014). The induction of the ER-derived eukaryotic pathway for chloroplast galactolipids upon heat stress has been reported in several species (Higashi and Saito, 2019). Furthermore, a previous report showed an increase of the ER-derived galactolipids and the key role of the ACYL-LIPID DESATURASE2 located in the ER in the acclimation to low temperature in *A. thaliana* (Chen and Thelen, 2013). The reported difference in the relative abundance of the galactolipid acyl chains supports the hypothesis that a shift from the plastid to the ER pathway for membrane lipid biosynthesis is part of the plant response to non-optimal temperatures. Considering the MGDG/DGDG ratio observed at 10°C, the relative DGDG content was higher than at 22°C. This is consistent with previous reports showing that, during acclimation to prolonged cold periods, the MGDG/DGDG ratio became smaller, while minor changes were observed in the concentration of the other major thylakoid membrane components PG and SQDG (Hendrickson et al., 2006). However, PG and SQDG may also have an impact on thylakoid membrane structure and dynamics depending on the desaturation of the acyl chains. In fact, PG accumulation and desaturation may be critical for photosynthetic acclimation to low temperature (Gao et al., 2015).

The differences in the galactolipid levels may affect the thylakoid organization. For instance, a larger DGDG fraction may strengthen the stacking of the thylakoid membranes by the interactions between facing headgroups of these galactolipids (Demé et al., 2014; Kanduè et al., 2017). However, a lower MGDG/DGDG ratio could also affect the stacking of the LHCII-rich portions of the thylakoid membranes (Seiwert et al., 2018). As the relative level of DGDG was higher at 30°C than in the other two growth temperature, it is plausible that the increased proportion of DGDG strengthens the membrane interactions. This, along with the lower average number of desaturations per molecule, may increase the thermal stability of the thylakoid membrane and may also have a direct effect on the NPQ component acting on the aggregation of LHCII (Krumova et al., 2010).

High temperature may directly affect the photosystem II electron transport efficiency and its structure causing the production of ROS (Pospíšil, 2016). Low temperature hampers PSII repair cycle, leading to photoinhibition and activation of ROS signaling pathways (Grimaud et al., 2013). Consistently, previous reports have shown that temperature changes lead to an increase in the concentration of antioxidant compounds embedded in the thylakoid membrane (Mène-Saffrané et al., 2010; Spicher et al., 2016). In a previous report on tomato, α-tocopherol and plastochromanol were shown to accumulate following a temperature increase, while minor to no change was observed in plants grown at low temperature (Spicher et al., 2016). This study, conversely, found a similar increase in α-tocopherol and plastochromanol concentrations in the plants grown at 10 and 30°C compared to the control condition at 22°C (Figure 5). It is interesting to note that PQ re-oxidation in the dark was faster in the plants grown at 10°C compared to the other two growth temperatures, as inferred by the chlorophyll *a* fluorescence parameter *V*_J_. In the absence of light, the oxidation of PQ is mostly dependent on the activity of the plastid terminal oxidase PTOX (Carol and Kuntz, 2001). In *A. thaliana* an accumulation of the PTOX protein was reported in plants acclimated to low temperature (Ivanov et al., 2012). However, the commercial antibody against PTOX detects multiple bands even on cress protein sample obtained from purified thylakoid (Supplementary Figure S5). Lacking any genomic data of *L. sativum*, it is not possible to exclude that different forms of PTOX exist in this species, although the majority of the sequenced higher plants contain a single PTOX gene (Nawrocki et al., 2015). These uncertainties do not allow drawing a conclusion on the accumulation of PTOX in cress at different temperatures. High level of PTOX protein was hypothesized to be responsible for an increase in the non-photochemical oxidation of PQ in the high mountain plant species *Ranunculus glacialis* as a strategy allowing a better acclimation to low temperatures combined with high irradiation (Streb et al., 2005). However, in this species low temperature alone was not sufficient to induce PTOX protein accumulation (Laureau et al., 2013). Furthermore, considering studies where an ectopic overexpression of the PTOX protein was achieved, it appears that the PTOX protein level is not the unique determinant of the non-photochemical PQ oxidation rate (Johnson and Stepien, 2016).

The crowded thylakoid membrane poses major limitations to the mobility of PQ and therefore may physically hamper its oxidation in the dark (Tremmel et al., 2003). Considering this, the differences in the galactolipid profile (Figure 5) and the change in the photosynthetic protein accumulation (Figure 4) observed at 10°C may be functional in facilitating PQ mobility and therefore its oxidation by PTOX (Pralon et al., 2020). Furthermore, the accumulation of plastochromanol, which appears to be tendentially higher at 10°C than at 30°C, would allow to contrast more efficiently membrane lipid peroxidation (Figure 5; Nowicka et al., 2013). Accumulation of lipid peroxides, in fact, would reduce the membrane fluidity and therefore the mobility of PQ (Niki, 2009). Accumulation of α-tocopherol, necessary to protect from ROS in both temperature condition, would, on the contrary, stabilize the membrane (Arora et al., 2000). The accumulation of γ-tocopherol, observed at 10°C (Figure 5A), may be a hint of a downregulation of α-tocopherol biosynthesis, functional to control its concentration and thus preserve the membrane fluidity. Membrane fluidity, and the mobility of PQ, may also explain the difference in qL observed between the plants grown at 10°C and those at 30°C (Figure 2). Lipid circulation would facilitate the access of oxidized PQ to PSII. The difference in the *V*_J_ kinetics in the dark between these two temperatures is consistent with a difference in long-distance PQ mobility (Figure 6). Increased fluidity may also facilitate the movement of PTOX to the grana stacks, as in *Eutrema salsugineum* under salt stress (Stepien and Johnson, 2018).

The presumed higher thylakoid membrane fluidity of the plants developed at 10°C compared to the other growth temperatures had, if any, a negative impact on PQ oxidation under far-red. Plants developed at 10°C displayed a slower photochemical PQ oxidation compared to 22°C. While in the plants grown at 30°C, PQ oxidation under far-red appears to be slightly faster than in those grown at 22°C (Figure 5B). This difference may be rationalized assuming that the accumulation of more saturated galactolipids and α-tocopherol observed at 30°C results in the stabilization of the thylakoid membrane structure and grana stacking (Arora et al., 2000; Krumova et al., 2010). In this scenario, the photochemical oxidation of PQ would be accelerated by the stabilization of PQ microdomains inside the grana allowing a more efficient circulation of PQ between PSII and Cytb_6_f (Kirchhoff et al., 2002). Conversely, the stabilization of the membrane may reduce PQ exchange between microdomains and between different portions of the thylakoid membrane, thus limiting the total PQ pool available per PSII and resulting in the lower ΦPSII and qL observed in the plants grown at 30°C (Figure 2). A hypothetic destabilization of the membrane domains, possibly due to the lower degree of galactolipid saturation, could explain the opposite effect on PQ mobility at 10°C compared to the 30°C scenario.

The dark re-oxidation rate, inferred from the *V*_J_ parameter, appears to be two order of magnitude slower than the one under far-red. In this scenario, the contribution of PTOX as an efficient alternative sink of electrons to protect the photosystems appears doubtful. Nevertheless, it has been shown that *Arabidopsis* plants lacking PTOX had a more severe variegation phenotype at lower temperature, suggesting that PTOX contribution is more critical at low temperature (Rosso et al., 2009). The increased non-photochemical oxidation of PQ may then serve to sustain the biosynthesis of carotenoids allowing to compensate for the enzymatic activity limitation imposed by the lower temperature (Norris et al., 1995). Consistently with this hypothesis, the accumulation of the most abundant carotenoids, on a dry weight basis, was only slightly lower in the plants grown at 10°C compared to the other conditions tested (Figure 3).

The differences observed in plants grown at higher and lower temperature are not symmetrical, but show some common differences, such as the higher level of the membrane antioxidants and a lower MGDG/DGDG ratio. However, at lower temperature, the acclimation is a trade-off between membrane fluidity and protection from ROS, which results in a detectable damage to PSII, while at higher temperature, membrane fluidity is not a constraint allowing to a more efficient protection. Furthermore, in the plants grown at lower temperature, the non-photochemical oxidation of PQ was faster compared to those at 22 and 30°C. This supports the model in which the activation of alternative electron pathways is an important strategy to protect the photosynthetic apparatus under cold stress.

## Data Availability Statement

The datasets generated for this study can be found in the Zenodo repository https://doi.org/10.5281/zenodo.3839245.

## Author Contributions

HS, ST, AG, LM, and PL designed the experimental plan. HS, ST, AG, LM, GB, and PL performed the experiments. GG performed the lipid profile analysis. ST and PL performed the statistical analysis of the data. PL wrote the manuscript. All authors read and approved the manuscript.

## Conflict of Interest

The authors declare that the research was conducted in the absence of any commercial or financial relationships that could be construed as a potential conflict of interest.

## Footnotes

1 http://www.psi.cz
