# Supplementary material for: Growth Temperature Influence on Lipids and Photosynthesis in Lepidium sativum
Source: Front Plant Sci. 2020 Jun 4;11:745. doi: 10.3389/fpls.2020.00745 (PMC7325982; doi:10.3389/fpls.2020.00745)
Supplement: Supplementary file 1 [file Data_Sheet_1.PDF]

MGDG 36:6

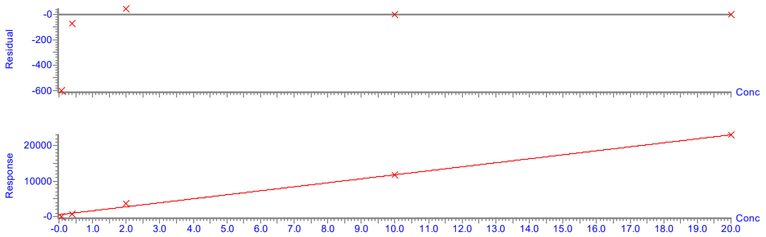

Standard concentration μg/ml

Calibration curve
1127.02*x + 528.47
Correlation coefficient
r^2 0.996

MGDG 34:6

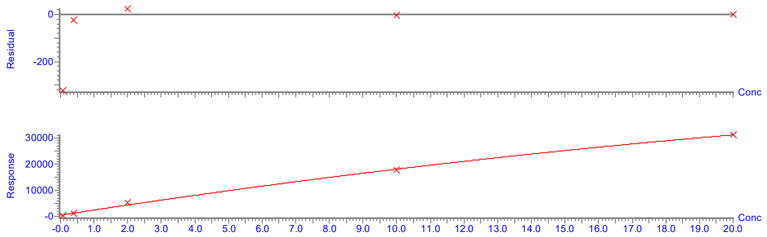

Standard concentration μg/ml

Calibration curve
$-22.57*x^2 +1984.11*x + 521.89$
Correlation coefficient
r^2 0.998

DGDG 36:6

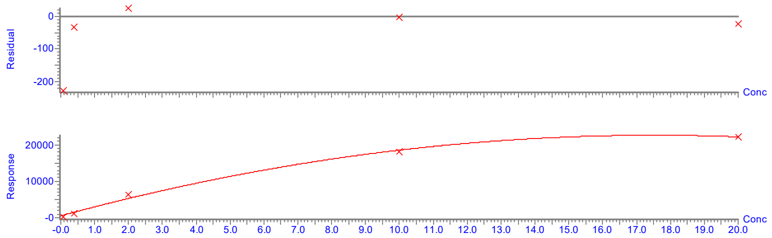

Standard concentration μg/ml

Calibration curve
$-71.87*x^2 +2520.08*x + 533.85$
Correlation coefficient
r^2 0.996

DGDG 34:6

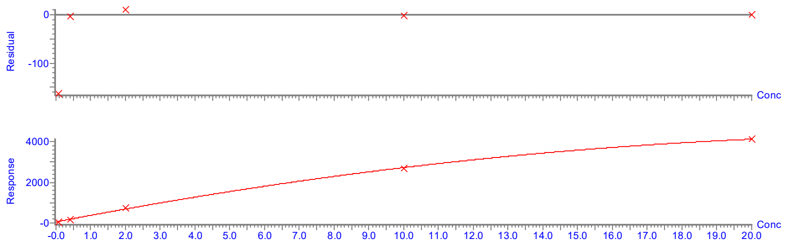

Standard concentration μg/ml

Calibration curve
$-6.39*x^2 +331.82*x + 52.75$
Correlation coefficient
r^2 0.999

Supplementary Figure 1. Calibration curves for galactolipids quantification.
The graphs show the correlation between the response, based on peak height, and the concentration of the standard mixture for the four molecules used for the quantification of the mono- (MGDG 36:6, MGDG 34:6) and di- (DGDG 36:6, DGDG 34:6) galactosyldiacylglycerols. Upper panel, residuals from the calibration; lower panel, measured points and calibration curve. The calibration was performed with TargetLynx XS (Waters) using a linear or a second-degree polynomial function. The equations of the functions and the correlation coefficients are shown on the right side of each graph.

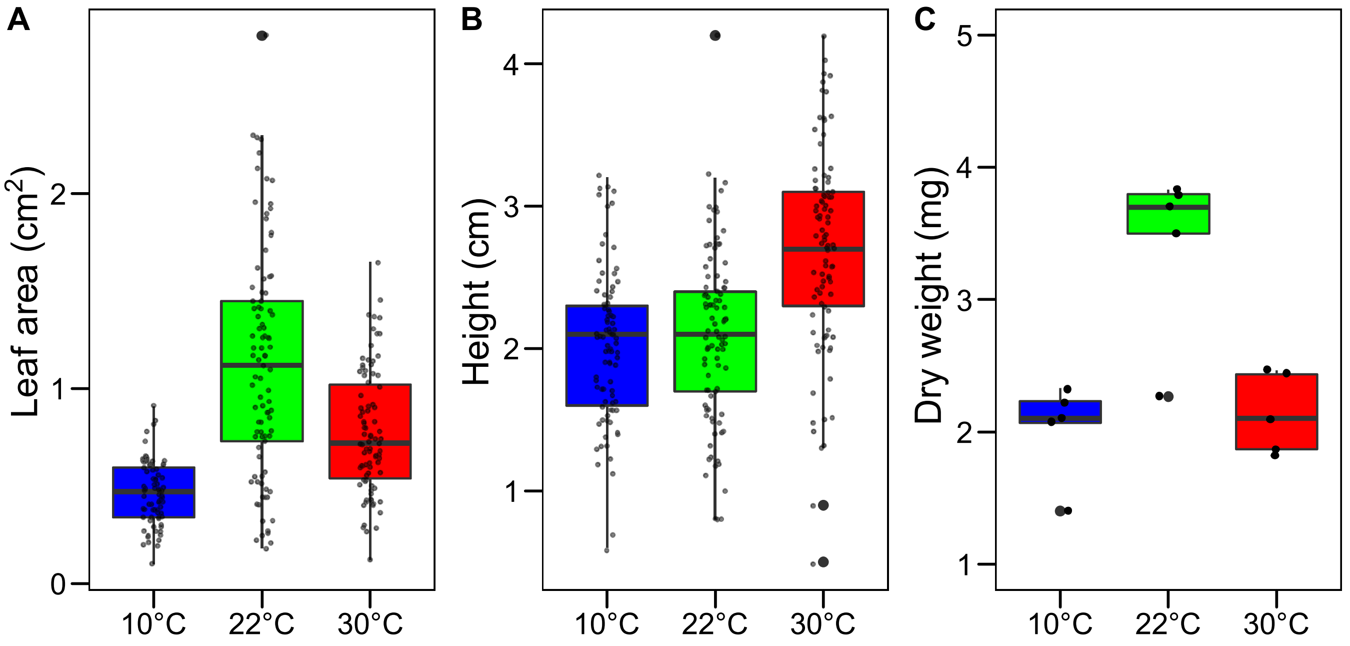

Supplementary Figure 2. Analysis of *L. sativum* grown at different temperatures

A) The average leaf area per plant was measured using ImageJ, based on the elaboration of a digital image taken for each pot and for each condition. The leaf area is significantly different for each growth condition ($p<0.0001$). **B)** Length of the hypocotyl for each individual was measured manually. The hypocotyl length of the plants grown at 30°C is significantly different from the other two conditions ($p<0.0001$). the same set of plants was used for the measure of these two parameters (10°C n=76, 22°C n=93, 30°C n=87). **C)** Average dry weight of a single cress plant after 120 hours of lyophilization, the data show the distribution of 5 samples for each growth temperature composed of three plants each, only the epigeal portion of the plant was considered. Both the plants grown at 10°C and at 30°C have a dry mass significantly lower than the control at 22°C ($p<0.005$). In the box plots, the lower and upper hinges correspond to the first and third quartiles (the 25th and 75th percentiles). The whiskers extends from the hinge to the farthest value no further than 1.5 times the distance between the first and third quartiles from the hinge. Farther points are considered as “outliers” and plotted individually.

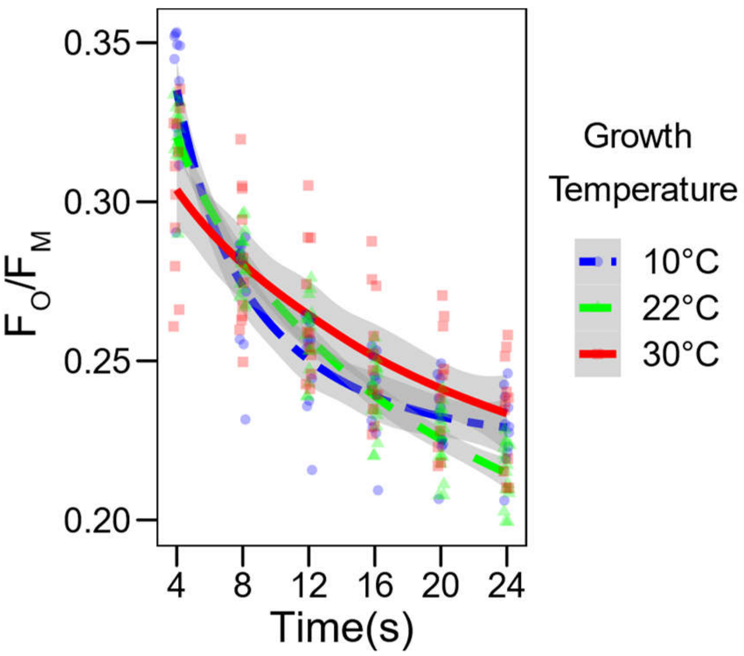

Supplementary Figure 3. Decrease of the Fo during dark incubation.

The Fo was calculated from the M-PEA (Hansatec) from the first pointe of the fluorescence rise during a a 700 ms saturating pulse for all the time points shown in Figure 6. Between the pulses the leaf was kept in the dark. . The points were interpolated with a logarithmic function (10°C, blue double dashed line, 22°C green dashed line, 30°C red continuous line). The standard error of the interpolation is shown as a grey area around the curve.

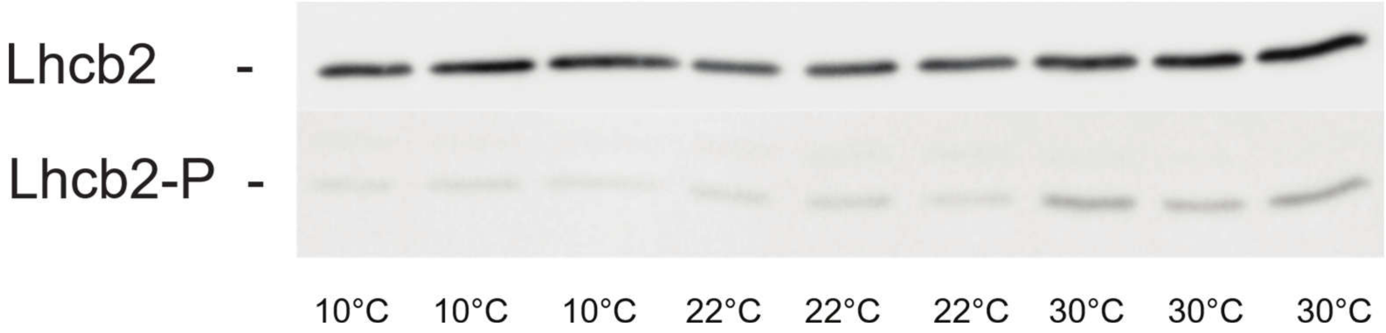

Supplementary Figure 4. Phosphorylation of Lhcb2 increases at higher temperature. in *L. sativum*
A membrane loaded with three protein samples prepared for different individuals grown at the same temperature (10°C, 22°C and 30°C) was decorated with the antybody against A) Lhcb2 and B) the phosphorylated form of the same protein (Lhcb2-P) (Agrisera AS13 2705). The position of the expected signal is shown on the left.

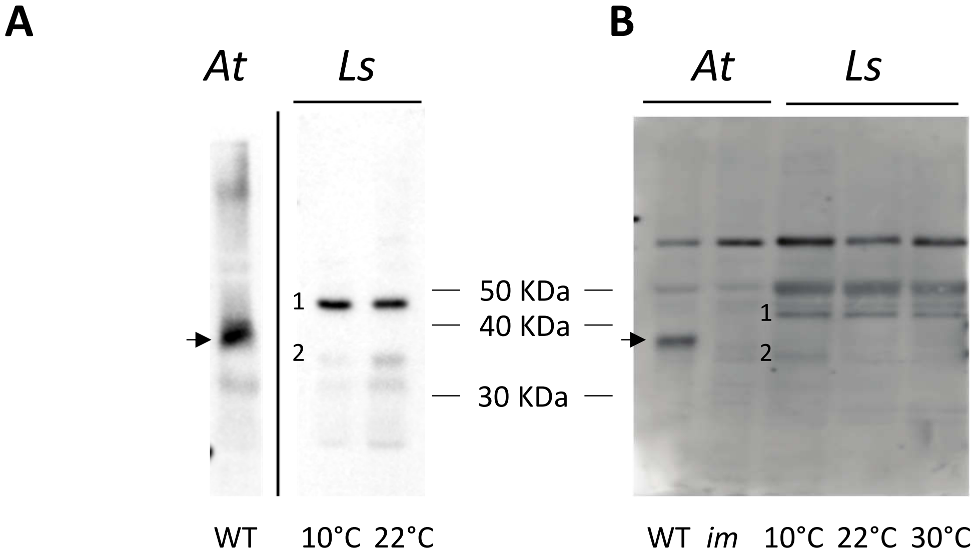

Supplementary Figure 5. Detection of the PTOX/im protein in *L. sativum*. Membranes containing extracts from *Arabidopsis thaliana* and *Lepidium sativum* were decorated with the commercial PTOX/im antibody (Agrisera) and revealed by ECL. A) The protein sample is derived from a thylakoid preparation from *Arabidopsis thaliana* (*At*) and *Lepidium sativum* (*Ls*) grown at 10°C and 22°C. The thylakoid preparation was performed according to Longoni et al. 2015. The samples were loaded by equal chlorophyll amount. Two differential bands were detected in the *L. sativum* samples, marked with 1 and 2, having a size consistent with the expected PTOX size (the average predicted size of the mature PTOX for the sequenced members of the Brassicaceae family is 35 KDa). The PTOX protein in the samples from *Arabidopsis thaliana* is highlighted with an arrow. B) representative immunoblot of the analysis of total protein extracts from *Arabidopsis thaliana* (*At*) and *Lepidium sativum* (*Ls*) grown at 10°, 22°C and 30°C. The mutant *im* (lacking the PTOX protein) was included to confirm the identification of the protein in *A. thaliana*. The bands 1 and 2 identified in the thylakoid preparation are shown in the total extracts according to their size.
